# Association between joint dislocation and malignant hyperthermia

**DOI:** 10.1002/anr3.70021

**Published:** 2025-07-09

**Authors:** A. O. Gomes, P. V. Andrade, J. M. Santos, L. S. Souza, A. S. B. Oliveira, M. Vainzof, H. C. A. Silva

**Affiliations:** ^1^ Department of Neurology Federal University of São Paulo São Paulo Brazil; ^2^ Department of Genetics and Evolutionary Biology Institute of Biosciences, University of São Paulo São Paulo Brazil; ^3^ Department of Anaesthesia Federal University of São Paulo São Paulo Brazil

**Keywords:** joint dislocation, malignant hyperthermia, myopathies, *RYR1*

## Abstract

Malignant hyperthermia is a potentially fatal autosomal dominant hypermetabolic pharmacogenetic syndrome resulting from altered intracellular calcium dynamics in skeletal muscle, triggered by halogenated anaesthetics and suxamethonium. Current evidence suggests a degree of association between malignant hyperthermia and joint dislocations. We evaluated 162 patients with a personal or family history of malignant hyperthermia utilising a standardised protocol. We found a significantly higher incidence of joint dislocations in patients with malignant hyperthermia compared to non‐susceptible patients (11% versus 0%, p = 0.002). This study contributes to understanding the long‐term clinical manifestations of malignant hyperthermia and consequently may help develop clinical management strategies which incorporate the risk of joint dislocations, such as care in positioning during anaesthesia, and therapeutic interventions to improve quality of life.

## Introduction

Joint dislocations are generally caused by acute traumatic injuries or repetitive strain. However, dislocations can be associated with connective tissue and muscle diseases, such as central core disease, which has been linked to the same *RYR1* gene involved in susceptibility to malignant hyperthermia (MH) [1, 2, 3, 4, 5, 6]. Malignant hyperthermia is a potentially fatal autosomal dominant genetic disorder triggered by halogenated volatile anaesthetics and the depolarising neuromuscular blocking agent suxamethonium. It is characterised by hypercarbia, tachypnoea, tachycardia, elevated body temperature, muscle rigidity, acidosis and rhabdomyolysis [5]. Predisposition to MH is most often associated with mutations in the *RYR1* gene, which encodes the type 1 ryanodine receptor, a calcium channel in the sarcoplasmic reticulum of skeletal muscles essential for regulating intracellular calcium homeostasis [6, 7]. Variants in the *CACNA1S* and *STAC3* genes have also been described [6].

Several authors from different countries have cited the presence of joint dislocations in patients with MH [8, 9, 10]. The description of joint dislocations in MH‐susceptible patients may indicate a possible association, either due to muscle alterations associated with MH or directly resulting from the genetic defect involved [9, 11]. Thus, we analysed the frequency of joint dislocations in MH‐susceptible patients at our MH centre.

## Methods

This study was approved by our Institutional Research Ethics Committee, according to the ethical guidelines established by the Declaration of Helsinki. Informed written consent was given by all participants. The study sample comprised all patients who presented with a personal or family history of MH, evaluated prospectively using a standardised protocol at an MH Reference Centre between January 2004 and December 2022. The evaluation included information on patient characteristics, clinical symptoms, personal and family medical history, neurological examination, serum creatine kinase levels, in vitro contracture test (IVCT) with halothane and caffeine according to the European MH Group protocol [5], muscle histochemistry and molecular studies. In addition, all patients were specifically questioned about the following signs and symptoms: exercise or heat intolerance, myalgia, cramps, muscle weakness, congenital malformations and osteoarticular conditions.

Among the osteoarticular conditions, we focused on reports of joint dislocation, objectively confirmed by clinical examination or imaging investigations. Additionally, patients with confirmed joint dislocation were invited for a second evaluation with a physical therapist, specifically focusing on the presence of joint hypermobility. Joint hypermobility was diagnosed by the Bulbena score when abnormal values (≥ 5 in women or ≥ 4 in men) were found.

The molecular studies used next‐generation sequencing to analyse 280 genes associated with neuromuscular disorders. Additionally, exome studies were conducted in one patient focusing on 36 genes known to be related to hypermobility disorders [12].

## Results

Among the 162 patients submitted to IVCT due to personal or family history of MH between 2004 and 2022, 90 were found to be MH‐susceptible based on positive result. Joint dislocation was present in 10 of the 90 MH‐susceptible patients and none of the 72 non‐susceptible patients (11% versus 0%, p = 0.0024). Details of the 10 patients with joint dislocations are available in Table 1. The 10 patients with joint dislocations were distributed across eight families and presented with dislocations predominantly in the lower limbs. Two patients had had a MH crisis, while the remaining eight reported a family history of MH. Three patients were relatives (family A: patients 1, 2 and 3) and reported that the MH‐index patient of their family also had patellar dislocation (index patient not evaluated in this study). Patient 5 (family C) reported 3 further relatives (also not evaluated) with patellar dislocation.

**Table 1:** Clinical characteristics of MH‐susceptible patients with joint dislocation.

Patient	Family	Age/sex	Joint dislocation	MH family history	Symptoms	Examination
1	A	51/F	Bilateral patellae	Yes	Exercise intolerance Myalgia	Muscle weakness Diminished reflexes
2	A	42/F	Bilateral patellae	Yes	Exercise intolerance Myalgia Cramps	Muscle weakness Scoliosis
3	A	35/F	Elbow	Yes	–	Muscle weakness Calf hypertrophy Micrognathia
4	B	32/M	Ankle	Yes	Gait delay Club‐feet	Ptosis Calf hypertrophy Micrognathia
5	C	23/F	Bilateral patellae	MH index patient	Exercise intolerance Myalgia Cramps Club feet	Muscle weakness Scoliosis Cavus feet Clinodactyly
6	D	43/M	Bilateral patellae	Yes	Exercise intolerance Cramps	Ptosis Diminished reflexes Muscle hypertrophy
7	E	29/M	Halux	Yes	Exercise intolerance Cramps	Muscle hypertrophy
8	F	23/F	Bilateral patellae	Yes	Myalgia	Ptosis Strabismus Scoliosis
9	G	32/F	Thumbs	Yes	Myalgia	Ptosis
10	H	22/F	Ankle Bilateral shoulders	MH index patient	Exercise intolerance Myalgia	Muscle weakness Ptosis Pectus carinatum

F, female; M, male; MH, malignant hyperthermia.

Malignant hyperthermia susceptible patients with joint dislocations exhibited other osteoarticular conditions, including scoliosis, micrognathia, pes cavus, clinodactyly and pectus carinatum and increased serum levels of creatine kinase were detected in 50%. Seven patients exhibited a positive IVCT on exposure to both halothane and caffeine (Table 2). Muscle histochemistry revealed abnormalities indicative of myopathy in three of the ten patients. Six different variants in the *RYR1* gene were found in eight patients, with four of them diagnostic for MH susceptibility (pathogenic/probably pathogenic). Four of the 10 patients with confirmed dislocation were available for the joint hypermobility assessment which detected abnormal results in three of them, with Bulbena scores of 8 and 9 in two women and 5 in one man. The remaining six patients were not available for this additional evaluation.

**Table 2:** Laboratory characteristics of MH‐susceptible patients with joint dislocation.

Patient	CK	IVCT	Muscle biopsy	Genetics	Variant classification
1	Normal	MHShc	Normal	*RYR1*: p.R614C	Pathogenic
2	Elevated	MHShc	Type 1 predominance	*RYR1*: p.R614C	Pathogenic
3	Normal	MHSh	Normal	*RYR1*: p.R614C	Pathogenic
4	Normal	MHShc	Normal	–	–
5	Elevated	MHShc	Myopathy	*RYR1*: p.Ile2182Phe	Uncertain significance
6	Elevated	MHShc	Cores	*RYR1*: p.Ala152Asp	Uncertain significance
7	Normal	MHSh	Normal	–	–
8	Normal	MHSh	Normal	*RYR1*: p.R2452G	Uncertain significance
9	Elevated	MHShc	Normal	*RYR1*:p.Gly2375Arg	Probably pathogenic
10	Elevated	MHShc	Normal	*RYR1*: p.Val4849Ile	Pathogenic

CK, creatine kinase; IVCT, in vitro contracture test; MH, malignant hyperthermia; MHSh, MH‐susceptible with contracture after halothane; MHShc, MH‐susceptible with contracture after caffeine and halothane; *RYR1*, ryanodine receptor type 1 gene.

## Discussion

This study suggests a higher frequency of joint dislocation may exist in MH‐susceptible patients compared to the general population. This finding is important to anaesthetists when caring for MH‐susceptible patients and their families. The presence of a family history of joint dislocation in two of the eight families of our sample could indicate a shared genetic predisposition which contributes to both joint dislocation and MH susceptibility. All patients with patellar dislocation in our sample had bilateral dislocation, a previously reported finding associated with a family history of joint dislocation [2]. While joint dislocation is more common in childhood than adulthood, the mean age of dislocation in our sample was in the fourth decade of life, which may be suggestive of a unique feature in MH‐susceptible patients [1].

Consistent with the existing literature, there was predominance in our sample of dislocations in the lower limbs, specifically of the patella, more common in women than in men [1, 2, 3, 13]. Other symptoms reported by patients in our cohort, such as exercise intolerance, myalgia and cramps, were similar to previous reports. Such symptoms have been related to the metabolic dysregulation which leads to an accumulation of calcium in muscle cells resulting in muscle stiffness during a crisis and chronic symptoms in daily life [14].

Mutations in the *RYR1* gene were identified in 80% of our MH‐susceptible patients with joint dislocations. The resulting dysfunction in the type 1 ryanodine receptor can lead to chronic abnormalities in muscle contraction and eventually myopathic changes, which may contribute to joint instability. However, our data indicated that the presence of muscle weakness was not always a prerequisite for joint dislocations in MH‐susceptible patients. Recently, Santos et al reported a higher frequency of joint hypermobility, which is a predisposing factor for joint dislocation, in MH patients without muscle weakness [15]. A direct deleterious action of dysfunctional type 1 ryanodine receptors on tendon cells is possible, contributing to joint hypermobility and dislocations [5]. Additionally, congenital deformities, such as micrognathia, clinodactyly, pectus carinatum and clubfoot, could be part of a broader spectrum of congenital syndromes which could be predisposing to joint dislocations [5].

Britt et al reported that MH‐susceptible patients may present with various musculoskeletal complications, including dislocations [7], while Taylor et al observed that members of a family with the c.7354C>T mutation in the *RYR1* gene frequently exhibited patellar dislocation, with some individuals requiring surgical intervention for stabilisation [10]. Early diagnosis and orthopaedic follow‐up of patients with joint dislocation are essential to prevent long‐term complications, such as osteoarthritis [16]. The presence of musculoskeletal problems is also of particular relevance to anaesthetists due to potential positioning injuries. Joint dislocations can also occur during airway management or performance of peripheral nerve blocks causing persistent nerve damage [17, 18, 19], and these risks may be exacerbated by the use of neuromuscular blockade due to the decrease in muscle tone.

Our study has limitations linked to the specific ethnic background of our population, which can differ from that of other countries, and our findings should be confirmed in more diverse samples of MH‐susceptible patients. Additionally, only a small number of MH‐susceptible patients suffered from musculoskeletal issues of joint dislocations in our sample, making it difficult to draw generalisations. As some MH‐susceptible patients live far from our MH centre and were evaluated only once, our study cannot rule out that they developed musculoskeletal problems throughout their lives, and thus our findings may be underestimated. A multicentre routine follow‐up of musculoskeletal problems in MH‐susceptible patients would be necessary to evaluate quality of life. This study contributes to the understanding of the long‐term clinical manifestations of MH susceptibility and the need to develop clinical management strategies which include regular assessment for joint dislocations, prevention of complications and care in patient positioning during anaesthetic procedures.
